# Evolutionary and Modern Image Content Differentially Influence the Processing of Emotional Pictures

**DOI:** 10.3389/fnhum.2017.00415

**Published:** 2017-08-23

**Authors:** Matthias Dhum, Uwe Herwig, Sarah Opialla, Michael Siegrist, Annette B. Brühl

**Affiliations:** ^1^Department of Consumer Behavior, Institute of Environmental Decisions, ETH Zurich, Switzerland; ^2^Department of Psychiatry, Psychotherapy and Psychosomatics, University Hospital of Psychiatry, University of Zurich Zurich, Switzerland; ^3^Department of Psychiatry and Psychotherapy III, University of Ulm Ulm, Germany

**Keywords:** emotion processing, fMRI, evolutionary content, fear module, amygdala

## Abstract

From an evolutionary perspective, environmental threats relevant for survival constantly challenged human beings. Current research suggests the evolution of a fear processing module in the brain to cope with these threats. Recently, humans increasingly encountered modern threats (e.g., guns or car accidents) in addition to evolutionary threats (e.g., snakes or predators) which presumably required an adaptation of perception and behavior. However, the neural processes underlying the perception of these different threats remain to be elucidated. We investigated the effect of image content (i.e., evolutionary vs. modern threats) on the activation of neural networks of emotion processing. During functional magnetic resonance imaging (fMRI) 41 participants watched affective pictures displaying evolutionary-threatening, modern-threatening, evolutionary-neutral and modern-neutral content. Evolutionary-threatening stimuli evoked stronger activations than modern-threatening stimuli in left inferior frontal gyrus and thalamus, right middle frontal gyrus and parietal regions as well as bilaterally in parietal regions, fusiform gyrus and bilateral amygdala. We observed the opposite effect, i.e., higher activity for modern-threatening than for evolutionary-threatening stimuli, bilaterally in the posterior cingulate and the parahippocampal gyrus. We found no differences in subjective arousal ratings between the two threatening conditions. On the valence scale though, subjects rated modern-threatening pictures significantly more negative than evolutionary-threatening pictures, indicating a higher level of perceived threat. The majority of previous studies show a positive relationship between arousal rating and amygdala activity. However, comparing fMRI results with behavioral findings we provide evidence that neural activity in fear processing areas is not only driven by arousal or valence, but presumably also by the evolutionary content of the stimulus. This has also fundamental methodological implications, in the sense to suggest a more elaborate classification of stimulus content to improve the validity of experimental designs.

## Introduction

Identifying threatening situations is a vital feature of human perception that has evolved over the history of animals. A failure in this aptitude could have had fatal consequences for our ancestors. The vital relevance of a working adaptive behavior is assumed to have led to the evolution of a dedicated fear module that, in turn, governs this behavior (Öhman and Mineka, 2001; Sander et al., 2003). Öhman and Mineka (2001) further argue that this module has evolved to recognize all natural threats facing our ancestors, such as predators and poisonous animals. The concept of the fear module is based on the theory of preparedness, which posits that the successful perception and identification of environmental threats lead to a reproductive advantage for the individual (Seligman, 1971).

In modern times, however, the environmental threats prevalent until a few centuries ago are no longer the main threats to most people, particularly in modern urban societies. Instead of the natural and evolutionary established threats we now increasingly face threats that are qualitatively different, more technical, and in some cases less tangible. This might require an adaptation of our perception, evaluation and reaction to these threats: We no longer have to fear for instance snakes, spiders and predators, but should rather be cautious in motor traffic, when facing guns as well as when handling tools like knives. These stimuli are in this study referred to as modern threats. The question is how the neural processes have adapted in response to these “newer” threats. In our study we therefore investigated the neural differences when comparing the perception of evolutionary threats to modern threats. In this line of investigation, we define the term “threat” as the anticipation of a spatially and temporally proximate source of potential harm for the individual (Baldwin, 1971; Davis et al., 2009). The concept of threat involves the identification of emotional significance, the generation of an affective state, and a subsequent behavior, they both engage overlapping neural structures and functions (Phillips et al., 2003; Mohr et al., 2010; Herwig et al., 2011). Earlier studies addressed the question of differences in the central nervous processing of evolutionary vs. modern threats (Blanchette, 2006; Fox et al., 2007; Brown et al., 2010; Sakaki et al., 2012). Regarding the threat-superiority effect, modern threats were reported to be detected in some instance better than evolutionary ones (Blanchette, 2006), whenever such a difference was not observed in an event-related potential study (Brown et al., 2010) or regarding reaction time (RT; Fox et al., 2007). Sakaki et al. (2012) reported differences regarding involved brain areas when comparing evolutionary and social stimuli with more activation in dorsomedial prefrontal areas in the social context.

The neural underpinnings of the perception of threats in general and associated negative emotions have been studied extensively with a range of methods and stimuli (LeDoux, 2000; Phan et al., 2002; Wager et al., 2003; Pessoa, 2008; Pessoa and Adolphs, 2010). The current model posits that a network of cortical and subcortical regions, including the amygdala, orbitofrontal cortex, anterior insula, anterior cingulate cortex, and inferotemporal visual cortex, play a central role in the perception and identification of threatening stimuli (Sabatinelli et al., 2005; Pessoa, 2008). While the amygdala was previously thought to be involved primarily in the perception of threatening (or more general, emotionally negative) stimuli, the concept of this subcortical region has progressed to a more general function of significance detection and processing (Sander et al., 2003; Williams, 2006; Pessoa and Adolphs, 2010). According to this concept, the amygdala should be activated when encountering any stimuli that convey a biological significance for the individual, which can be of either positive or negative valence (Sergerie et al., 2008). Neuroimaging studies support this assumption by showing that amygdala activity varies according to the level of arousal evoked by a stimulus (Kensinger and Corkin, 2004; Sabatinelli et al., 2005; Kensinger and Schacter, 2006; Kryklywy et al., 2013). However, valence, which is the other main dimension in the Circumplex model of affect (Russell, 1980), seems to have a smaller effect on amygdala activity (Phan et al., 2002; Wager et al., 2003; Sergerie et al., 2008).

To investigate the influence of content on the neural circuits involved in processing threatening stimuli, we chose pictures showing a different phylogenetic origin by selecting those with a strong evolutionary history vs. modern pictures. As a reference, we included two neutral categories, again comprising evolutionary prepared vs. modern pictures. Thus, our study included four experimental conditions: evolutionary-threatening, modern-threatening, evolutionary-neutral and modern-neutral. The pictures included in our study displayed threatening stimuli related to the basic emotion of fear. In contrast, pictures showing disgust and sadness were not covered in our study.

On a neurophysiological level, we propose that the affective pictures will engage a network of brain regions comprising amygdala, orbitofrontal cortex, anterior insula, anterior cingulate cortex, inferotemporal visual cortex as well as medial thalamus and midbrain (Sabatinelli et al., 2005). We consider two complementary lines of reasoning which serve as a theoretical frame in our study. First, literature (Sander et al., 2003; Wager et al., 2003; Sergerie et al., 2008) suggests that the neural activity in emotion processing circuits reflects the affective rating of the International Affective Picture System (IAPS) pictures—especially the arousal dimension, and to a lesser extent the valence dimension. Second, the theory of the evolved fear module (Öhman and Mineka, 2001) suggests differences between evolutionary and modern stimuli in the activation of emotion processing circuits. The evolution of the fear module in response to threats such as snakes and spiders implies that evolutionary threatening stimuli might be associated with a stronger activation particularly in evolutionary older regions as amygdala, thalamus and midbrain than the modern stimuli, which are supposed to evoke stronger activation in cortical stimulus processing areas as inferotemporal cortex.

## Materials and Methods

### Subjects

We recruited healthy subjects through a mailing list and pin board postings. Exclusion criteria were any history of major medical conditions, head trauma, neurological and psychiatric disorder (both individually and in the family), current substance abuse and medication; further contraindications against MRI such as claustrophobia, pregnancy, pace maker or ferromagnetic implants. These criteria were assessed in a semi-structured clinical interview. Subjects received CHF 50 compensation.

In total, 44 subjects (22 females) were scanned for the study. Three subjects were excluded from the final analysis (two subjects due to performance in the behavioral task suggesting a lack of attention or cooperation or otherwise misunderstanding of the instructions (RT in 35% of the trials >1.5 s or button presses outside the required time frame) and one subject due to potential clinical conditions which the subject revealed only after inclusion). Thus, the final sample comprised 41 subjects (21 females) with an average age of 25.0 years (SD = 5.3 years).

All subjects had normal or corrected-to-normal vision and were right-handed according to the Annett handedness questionnaire (Annett, 1967). All subjects were within the normal range of anxiety according to the State-Trait Anxiety Inventory X1 and X2 (Spielberger et al., 1970; Laux et al., 1981). No subject reported phobic symptoms related to the stimulus material (e.g., arachnophobia).

The study was approved by the Ethics Committee of the Canton of Zurich (Kantonale Ethikkommission Zürich^1^). All subjects gave their written informed consent. The study was conducted in accordance to the Declaration of Helsinki (World Medical Association, 2008).

### Stimulus Material

For each of the four experimental conditions, we selected 16 representative pictures from the IAPS database (Lang et al., 2008). First, the pictures were assigned to the respective condition based on a content analysis. This was done independently by two of the authors (MD, ABB) and discussed with the co-authors in case of divergent assignments. Second, the assignment was based on the ratings provided by the IAPS technical report (Lang et al., 2008). Each picture condition was constructed with the aim to not contain outliers in the valence and arousal ratings. Thus, pictures were selected for a condition if their rating was homogeneous within the condition and distinct to the other conditions. This manual selection process was validated in a pre-test with an independent larger sample (*N* = 201) by running a confirmatory factor analysis across all pictures (unpublished data). The evolutionary-threatening condition included pictures of predatory animals (e.g., snakes, spiders, dogs, bears, sharks) whereas the modern-threatening condition displayed pictures of guns, knives, and accidents involving cars, ships and airplanes. Evolutionary-neutral pictures comprised landscapes, forests and flowers, while the modern-neutral pictures showed inanimate objects such as cars, trains, ships, bridges, suitcases, and drawers. Picture numbers are provided in the Supporting Information, Supplementary Table S1.

### Experimental Procedure

**Figure 1:**
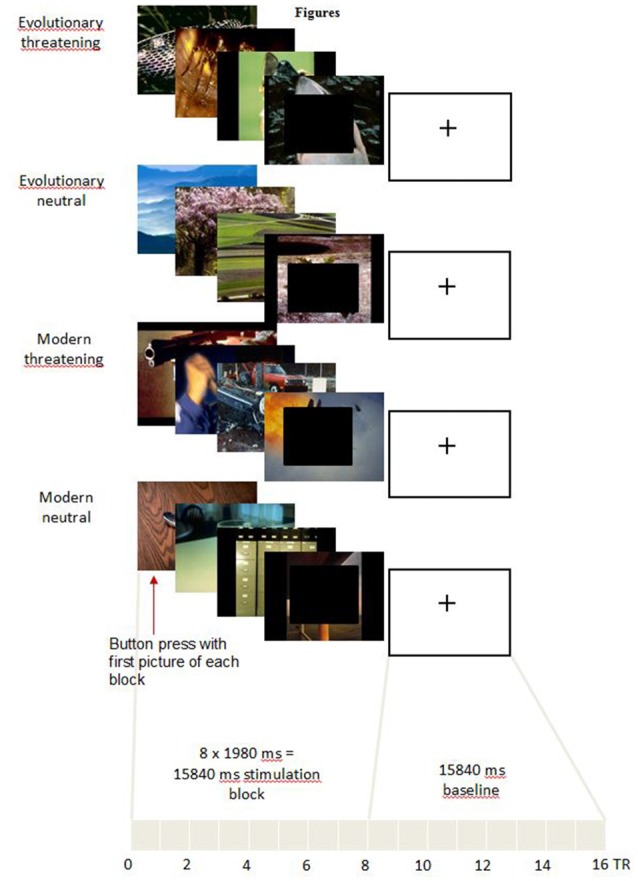
Experimental task. For representational reasons, only four pictures for each category are shown. In the experiment, each block consisted of eight pictures. In order to make the pictures less identifiable in the sense of the International Affective Picture System (IAPS) providers, in the figure black boxes are pasted over the front picture which of course was not the case in the experiment.

In the scanner, pictures were displayed covering the full screen of digital video goggles (Resonance Technologies, Northridge, CA, USA) using Presentation software (version 15.1^2^). We presented blocks of eight consecutive pictures from the same experimental condition (Figure 1). Each picture was shown for 1980 ms. Thus, each block lasted 15,840 ms in total. Before the first block and between the blocks, a black screen with a white fixation cross was shown for 15,840 ms to allow the Blood-Oxygen Level-Dependent (BOLD) signal to return to a baseline (Ogawa et al., 1990).

The pictures for each block were randomly taken from the 16 pictures selected for the respective experimental condition. The block order was pseudo-randomized across an experimental run to control for serial position effects. One experimental run included four blocks of each experimental condition. Thus, each of the 16 pictures of every experimental condition was shown twice in an experimental run. The whole experiment consisted of three experimental runs, each lasting approximately 11 min.

The subjects were instructed to press the button of a response box with their right index finger at the onset of the first picture of a new block. The recorded RT served as a control for general attention and wakefulness of the subjects. Further, fast RT are generally associated with higher fear relevance of the stimulus (Fox et al., 2007). After the scanning session, subjects rated the pictures on the valence (scaled from 1 = negative to 9 = positive) and arousal (scaled from 1 = low to 9 = high) scales using a digital version of the original IAPS self-assessment manikin (Mogg et al., 1994).

Similar to previous studies (Anders et al., 2008), we deliberately decided against an online rating during the functional magnetic resonance imaging (fMRI) task since it has been shown that emotional rating instructions may influence neural activity already during the perception of a stimulus (Taylor et al., 2003). Moreover, the post-scan evaluation of stimuli has been demonstrated to correspond well with the emotional experience during the scan (Hariri et al., 2000; Phan et al., 2004).

### Behavioral Data

We removed outliers (RT <100 ms or >1500 ms) from the data gathered during the scan. A repeated-measures ANOVA was performed to check for differences in RT to the different experimental conditions. In case of significant Mauchly’s tests of sphericity, Greenhouse-Geisser correction was applied. Bonferroni-corrected *post hoc* tests were performed to reveal differences between single conditions. Similarly, repeated-measures ANOVAs and subsequent *post hoc* tests were performed to test for differences in valence and arousal ratings between experimental conditions. Statistical analysis was performed with SPSS (Version 19.0.0.1, SPSS Inc., Chicago, IL, USA) and Matlab (Version R2014a; The MathWorks Inc., Natick, MA, USA).

### Image Acquisition

Imaging was performed using a 3.0 T GE Signa HD Scanner (GE Medical Systems, Milwaukee, WI, USA; 8-channel head coil). fMRI was conducted using echo-planar imaging (EPI) with the following configuration: 28 interleaved axial slices, 3.5 mm slice thickness, 0.5 mm gap, matrix 64 × 64, 240 mm field of view, resulting voxel size 3.75 × 3.75 × 4.0 mm, repetition time (TR) = 1980 ms, echo time (TE) = 32 ms, flip angle = 70°. The slice angle was optimized to reduce susceptibility artifacts in the amygdala and frontal regions. Per run a total of 328 volumes were acquired, 16 for each of the 20 experimental blocks. The first four volumes of each run were discarded to allow for T1 equilibration. In addition, 3-D T1-weighted anatomical volumes (172 axial slices, TR = 9.9 ms, TE = 2.9 ms, matrix size 256 × 256, voxel size 1 × 1 × 1 mm) were acquired for co-registration with the functional data. Furthermore, T2-weighted images in parallel to the EPI sequence were acquired to exclude possible T2-sensitive brain abnormalities.

### Image Analysis and Statistics

Imaging data was analyzed using BrainVoyager QX 2.8.4 (Brain Innovation, Maastricht, Netherlands; Goebel et al., 2006). Pre-processing of the functional data included slice scan time correction, 3-D motion correction with intra-session alignment, and temporal high-pass filtering with removal of linear trends. Functional data was co-registered to the individual anatomical 3-D datasets. Anatomical datasets were corrected for intensity inhomogeneity and transformed into Talairach coordinate space (Talairach and Tournoux, 1988). Volume time courses with a 3 × 3 × 3 mm^3^ voxel size were created from the functional datasets. For the subsequent group analysis, the volume time courses were spatially smoothed with a 6.0 mm full-width at half-maximum Gaussian kernel.

The experimental conditions were used as HRF-convolved box-car function predictors in the General Linear Model (GLM) design matrix. In addition, the individual 3-D motion correction parameters were z-transformed, high-pass filtered (10 cycles) and linear detrended using the BVA Predictor Tool (Version 1.52, J.M. Born, Maastricht, Netherlands), and added as predictors of no interest to the design matrix to account for BOLD artifacts caused by task-correlated motion (Morgan et al., 2007). From the individual GLM matrices, we calculated a Random Effects GLM as a first step in the group analysis. Voxel time courses from the single runs were percent-transformed. Serial correlations were detected and removed using the AR(2) model approach. We automatized most pre-processing steps using BrainVoyager scripts or WinAutomation software (Version 4.02, Softomotive Ltd., Athens, Greece).

Our aim was to analyze the differential activation of those brain regions centrally involved in the processing of negative emotional stimuli. In a first step, we identified brain regions activated by all threatening stimuli compared to neutral stimuli. Therefore, we calculated a repeated measures 2 × 2 ANOVA with the factors threat (levels: threatening, neutral) and origin (levels: evolutionary, modern). The voxel-wise threshold for statistical maps correspond to *p* < 0.001 uncorrected. To correct for multiple comparisons, a Monte Carlo simulation with 1000 iterations was used for estimating cluster-level false-positive rates on these maps (statistics implemented in BrainVoyager). This resulted in a minimum cluster size of 34 voxels at 3 × 3 × 3 mm (904 mm^3^), corresponding to *p* < 0.05 corrected cluster-wise.

In a second step, we analyzed the differential effect of the factor *origin* in the threatening stimuli. Therefore, we created individual maps for the two contrasts (evolutionary-threatening > evolutionary-neutral) and (modern-threatening > modern-neutral). These individual maps were subsequently used as input for a paired *t*-Test where we contrasted the maps “evolutionary-threatening > evolutionary-neutral” and “modern-threatening > modern-neutral” against each other. The voxel-level threshold for statistical maps corresponds to *p* < 0.001. To correct for multiple comparisons, a Monte Carlo simulation with 1000 iterations was used for estimating cluster-level false-positive rates on these maps. This led to a minimum cluster size of 38 voxels at 3 × 3 × 3 mm (1007 mm^3^), corresponding to *p* < 0.05 corrected cluster-wise. Further, we extracted *t* values from the resulting clusters to quantify the effect of *origin*. In selected regions, we additionally computed for each condition the mean time course by averaging all peri-stimulus BOLD time course segments belonging to the same condition using the respective tool in BrainVoyager. Anatomical regions were identified using the Talairach Client (Lancaster et al., 2000).

## Results

### Behavioral Results

We performed a repeated-measures ANOVA to test for differences in RT to the first picture of a block. Mauchly’s test indicated that the assumption of sphericity had been met ($\chi_{\text{(5)}}^{\text{2}}$ = 7.00, *p* > 0.05). The results showed no significant effect of experimental condition on RT (*F*_(3,120)_ = 1.58, *p* > 0.05). Mean RT ranged from 542.49 ms to 561.56 ms (Table 1).

**Table 1:** Reaction times to the first picture of a block during the scan session, and means and standard deviations of the normative ratings of the International Affective Picture System (IAPS) pictures.

	Reaction time (ms)	Valence	Arousal
Condition	*M (SD)*	*M (SD)*	*M (SD)*
Evolutionary-threatening	546.30 (175.31)^a^	3.93 (0.21)^a^	4.97 (0.25)^a^
Modern-threatening	542.49 (154.88)^a^	2.78 (0.17)^b^	5.07 (0.23)^a^
Evolutionary-neutral	556.74 (167.37)^a^	7.47 (0.11)^c^	2.21 (0.19)^b^
Modern-neutral	561.56 (165.70)^a^	5.35 (0.12)^d^	1.95 (0.15)^b^

*Valence scale from 1 = negative to 9 = positive and arousal scale from 1 = low to 9 = high. Non-identical superscripts a–d indicate conditions that are significantly different at *p* < 0.05 regarding comparisons of reaction times and valence vs. arousal. Reaction times to the first picture of a block were recorded during the fMRI scan, valence and arousal ratings after the scan*.

The general pattern of the post-scan rating of valence and arousal of the IAPS pictures did not deviate from the original ratings provided in the IAPS technical report (Lang et al., 2008) and from our own data in an independent sample (unpublished data). A confirmatory factor analysis in this independent sample on the valence and arousal ratings supported the assignment of the pictures to the four conditions, thus adding to the validity of the experimental design.

To test for differences in the valence rating between experimental conditions, we conducted a repeated-measures ANOVA. Mauchly’s test was significant ($\chi_{\text{(5)}}^{\text{2}}$ = 28.75, *p* < 0.05), indicating a violation of the sphericity assumption. Greenhouse-Geisser corrected values showed significant differences between experimental conditions (*F*_(2.11,84.40)_ = 222.76, *p* < 0.05). Bonferroni-corrected *post hoc* tests revealed significant differences for all pairwise comparisons between all conditions at *p* < 0.05 (Table 1).

Differences in the arousal rating between experimental conditions were assessed with a repeated-measures ANOVA. Mauchly’s test was significant ($\chi_{\text{(5)}}^{\text{2}}$ = 26.14, *p* < 0.05), indicating a violation of the sphericity assumption. Greenhouse-Geisser corrected values showed significant differences between experimental conditions (*F*_(2.02,80.79)_ = 119.36, *p* < 0.05). Bonferroni-corrected *post hoc* tests indicated that each threat condition was rated significantly different to both neutral conditions at *p* < 0.05 (Table 1).

To summarize, evolutionary-neutral pictures were rated significantly more positive in valence compared to all other conditions and within the positive spectrum of the IAPS set. Subjects rated both threat conditions significantly higher in arousal and more negative in valence than the two neutral conditions. While the two threatening conditions did not differ in arousal (*p* > 0.5), modern-threatening pictures were rated significantly more negative in valence than evolutionary-threatening pictures. Across all presented pictures, the subjects’ ratings varied significantly more on the arousal scale than on the valence scale (*N* = 64 pictures; average SD across pictures: valence = 1.39, arousal = 1.77, Wilcoxon *Z* = −5.5, *p* < 0.001).

### fMRI Results

**Figure 2:**
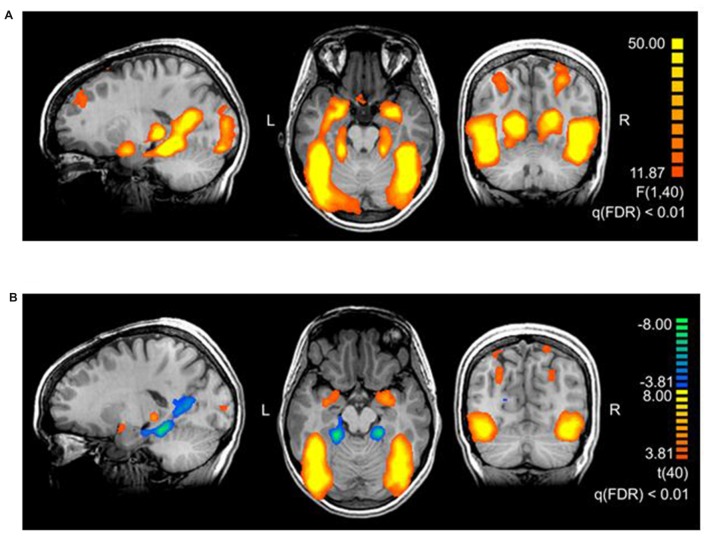
**(A)** Brain areas activating stronger for threatening stimuli than for neutral stimuli. The map shows the main effect of threat, derived from a repeated measures 2 × 2 ANOVA with factors threat (levels: threatening, neutral) and origin (levels: evolutionary, modern). The thresholds in the figures are chosen for representational purposes, *q*(FDR) < 0.01. Talairach coordinates of slices x: 18, y: −56, z: −17. **(B)** Brain areas showing the differential effect of origin in threatening pictures. Contrast: (Evolutionary-threatening > Evolutionary-neutral) > (Modern-threatening > Modern-neutral), q(FDR) < 0.01. Talairach coordinates of slices x: −18, y: −66, z: −12.

The main effect of *threat* in the 2 × 2 repeated measures ANOVA (factors *threat* and *origin*) revealed a network of cortical and subcortical regions (Figure 1) including the left middle frontal gyrus, right inferior frontal gyrus, right posterior cingulate gyrus, right cuneus, large portions of the bilateral occipital lobe including extrastriate and inferotemporal regions, and bilateral amygdala (see Table 2, Supplementary Table S2 and Figure 2A).

**Table 2:** Anatomical regions activating stronger for threatening stimuli than for neutral stimuli.

	Talairach coordinates				
Region (BA)	*x*	*y*	*z*	Cluster size (mm^3^)	*F*_peak_
L middle frontal gyrus (9)	−40	13	24	197	14.31
R inferior frontal gyrus (46)	35	31	12	423	17.62
R posterior cingulate gyrus (31)	8	−38	30	893	16.37
R posterior cingulate gyrus (29)	9	−50	12	1384	42.74
R cuneus (17)	8	−80	9	4742	54.31
L occipital lobe, extending into the inferior temporal lobe (18, 19, 37)	−43	−80	−9	54,405	99.61
R occipital lobe, extending into the inferior temporal lobe (18, 19, 37)	25	−32	−15	47,724	75.84
L amygdala	−25	1	−15	1069	20.67
R amygdala	20	−2	−12	1145	21.37

*Statistical threshold: *p* < 0.05 (cluster level corrected for multiple comparisons using a Monte Carlo simulation with 1000 iterations to estimate cluster-level false-positive rates). Abbreviations: L, left hemisphere; R, right hemisphere; BA, Brodmann area; Talairach coordinates of peak voxel, F values of peak voxel for the ANOVA. For MNI coordinates consider Supplementary Table S2*.

To identify regions showing a differential activation to the evolutionary vs. modern *origin* within the threatening stimuli, we applied the combined contrast “evolutionary-threatening > evolutionary-neutral” > “modern-threatening > modern-neutral”. This analysis revealed a network of regions including the left inferior frontal gyrus, right middle frontal gyrus, right parietal lobe (sub-gyral), right precuneus, left thalamus, bilateral fusiform gyrus, bilateral superior parietal lobule, bilateral amygdala (see Table 3, Supplementary Table S3 and Figure 2B).

**Table 3:** Anatomical regions showing the differential effect of origin in threatening pictures.

	Talairach coordinates				
Region (BA)	*x*	*y*	*z*	Cluster size (mm^3^)	*t*
L inferior frontal gyrus (9)	−39	14	25	4022	5.67
R middle frontal gyrus (9)	36	11	25	623	5.21
L fusiform gyrus (19)	−39	−82	−11	35,262	13.36
R fusiform gyrus (19)	39	−58	−14	31,408	12.46
L superior parietal lobule (7)	−24	−67	37	2195	5.16
R superior parietal lobule (7)	21	−58	67	673	5.90
R parietal lobe (sub-gyral) (7)	24	−52	52	400	4.93
R precuneus (7)	27	−73	43	1827	5.51
L amygdala	−30	−4	−14	1197	5.74
R amygdala	24	−1	−11	2815	6.42
L thalamus	−21	−28	1	434	6.19
L posterior cingulate (30)	−21	−55	13	2244	−6.20
R posterior cingulate (30)	18	−31	−13	1641	−7.15
L parahippocampal gyrus (36)	−21	−40	−8	3359	−8.79
R parahippocampal gyrus (35)	21	−34	−11	1775	−8.04

*Two-sample t-Test, contrast (Evolutionary-threatening > Evolutionary-neutral) > (Modern-threatening > Modern-neutral). All t-Tests significant at *p* < 0.001. Abbreviations: L, left hemisphere; R, right hemisphere; BA, Brodmann area; Talairach coordinates of peak voxel. For MNI coordinates consider Supplementary Table S3*.

**Figure 3:**
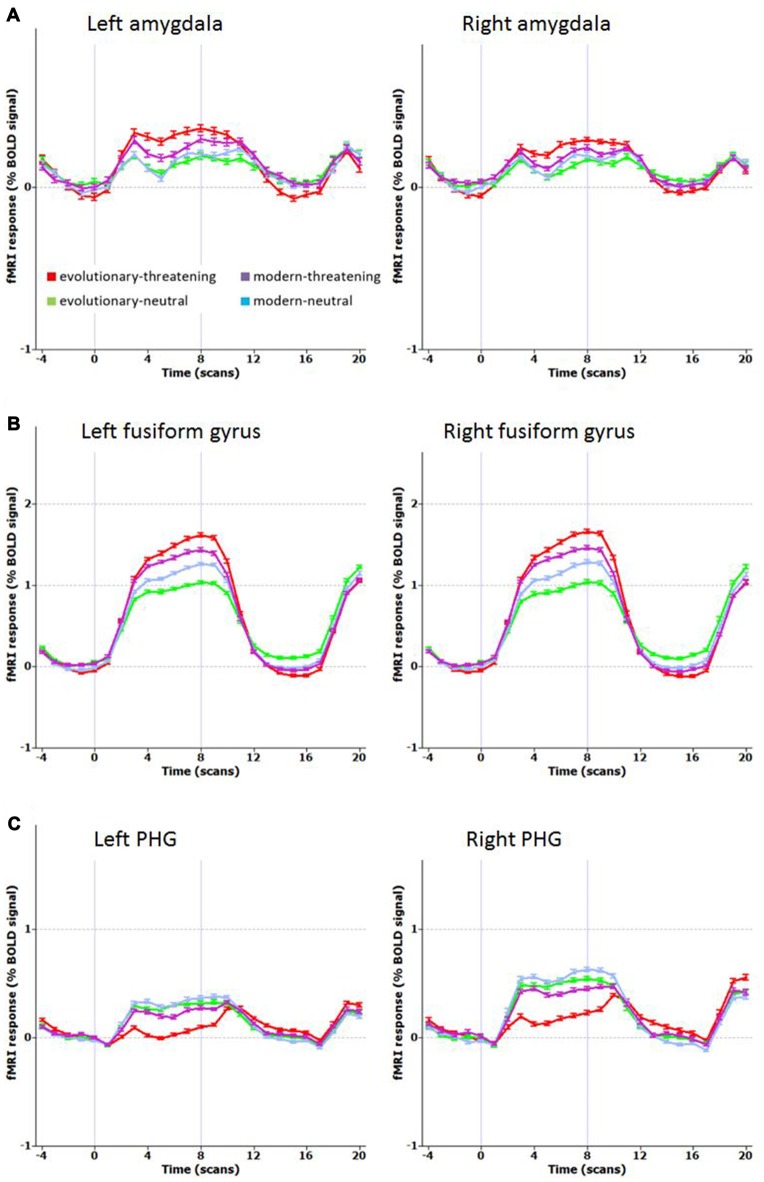
Mean time course for selected regions. **(A)** Left and right amygdala, **(B)** Left and right fusiform gyrus (BA 19), **(C)** Left and right parahippocampal gyrus PHG, (BA 36 left, BA 35 right). Contrast: (Evolutionary-threatening > Evolutionary-neutral) > (Modern-threatening > Modern-neutral).

The opposite contrast revealed that only in the bilateral posterior cingulate and the bilateral parahippocampal gyrus the activity was higher for modern-threatening stimuli than for evolutionary-threatening pictures (see Table 3). For the amygdala, the fusiform gyrus and the parahippocampal gyrus, we created event-related averages of all conditions to characterize the BOLD response of each experimental condition (see Figure 3).

## Discussion

### Functional Implications

We systematically investigated the effect of image content in threatening stimuli on the activation of neural networks involved in emotion processing. By contrasting threatening with neutral pictures, we revealed a network of regions typically found in emotion processing (Pessoa and Adolphs, 2010), thus supporting the validity of our threatening stimuli. Evolutionary-threatening pictures evoked significantly stronger activations than modern-threatening pictures in most regions of the network for processing threatening stimuli. Surprisingly, however, this finding is in contrast to the behavioral part of the experiment, the post-scan rating of the IAPS pictures. Subjects rated modern-threatening stimuli as significantly more negative in valence than evolutionary-threatening pictures, indicating a higher level of perceived threat or fear for stimuli such as guns, knives and car accidents. At the same time, the two threatening conditions did not differ in the arousal rating, thus implying no relevant association of subjective arousal with the difference of neural activity between the threatening conditions. According to the prevalent opinion in the literature, our behavioral findings would have suggested that modern-threatening pictures evoke a stronger BOLD response than the evolutionary-threatening pictures in regions involved in emotion processing, or the fear module, respectively (see Sabatinelli et al., 2005; Kensinger and Schacter, 2006). However, since the opposite was the case in our study, we argue that the evolutionary preparedness of the evolutionary-threatening stimuli is the actual driver of the neural activity.

The network of brain regions that activated stronger for evolutionary-threatening stimuli than for modern-threatening stimuli comprised bilateral amygdala, the left inferior frontal gyrus, right middle frontal gyrus, right parietal lobe (sub-gyral), right precuneus, left thalamus, bilateral fusiform gyrus and bilateral superior parietal lobule (Sabatinelli et al., 2005). The finding in the amygdala as central emotion processing region supports the close relationship to emotion processing (Figure 3A), but also early region in the visual stream as fusiforme gyrus, known for face processing (Sabatinelli et al., 2005), are involved (Figure 3B). We found the opposite effect (higher activity for modern-threatening than for evolutionary-threatening stimuli) in the bilateral posterior cingulate and the bilateral parahippocampal gyrus (Figure 3C). A possible explanation of this reversal could be the connection of the posterior cingulate gyrus with the hippocampus (FeldmanHall et al., 2016). Modern stimuli might engage more processes of memory, self-reflection and appraisal, which could be mediated by the posterior cingulate cortex. The parahippocampal gyrus has been found to encode complex visual scenes and the local environment (Epstein and Kanwisher, 1998). This could explain the low activation in this area for evolutionary-threatening stimuli, where the focus is on the animal itself and not so much on its surroundings (see Figure 3). In the other experimental conditions, however, about half of the pictures show wide-angled shots of natural landscapes or built environments, thus possibly activating the parahippocampal place area (Aguirre et al., 1996; Epstein and Kanwisher, 1998; Ishai et al., 2004).

The results of our study support the hypothesis of the amygdala and its connected regions as an evolved module for the detection of threat. This detection takes place automatically, without the need of cognitive processing of the stimuli (Lundqvist and Ohman, 2005). Furthermore, our results stress the perceived biological significance of evolutionary prepared stimuli, even if they do not pose such an actual threat to the individual anymore. A recent study found neurobiological evidence for a rapid snake detection mechanism in the pulvinar, which could represent a part of the evolved module (Stuber et al., 2011).

Our findings suggest that the assumption of amygdala activity explained by arousal ratings may not be fully comprehensive. Also, at least in our study, the valence ratings do not seem to reflect the activation of the emotion network. The study by Anders et al. (2008) showed effects of valence in line with the majority of the literature (i.e., less neutral valence ratings correlating with higher amygdala activity). However, the most negative rated pictures (modern-threatening) interestingly did not show the highest amygdala activity. When investigating amygdala activity to threatening stimuli, the explicit arousal and valence ratings might not be the strongest indicators to predict the neural activity. Even when made quickly and intuitively, these ratings might comprise elaborate cognitive evaluations and might thus not be strongly indicative of the amygdala’s role of automatic significance detection.

Also, alternative explanations of the difference in activation between the evolutionary-threatening and the modern-threatening condition can be taken into account. First, a complimentary explanation for the diminished BOLD response towards the modern-threatening stimuli could lie in a cognitively more demanding evaluation after the perception. Since an evolved module for these modern pictures can hardly exist, the evaluation of threat might require higher-level cortical processing, which in turn reduces amygdala activity (Hariri et al., 2000, 2003). This demanding evaluative process might set in involuntarily even in the absence of an additional experimental task.

Second, the conditions could potentially differ in perceived threat, when assuming that threat could not be defined by valence and arousal ratings. Some of the pictures display an immediate threat (e.g., snarling snakes and pointing guns), whereas other pictures only show a distal or already occurred threat (e.g., resting spiders or car accidents). It might be possible to quantify the threat potential of either experimental category in terms of probabilities, for instance by pooling lethality rates of each stimulus displayed. However, we assume that the individual rating of valence and arousal represents an appropriate and valid proxy to the subjective feeling of threat. Moreover, by averaging across a broad range of image content, we reduce the influence of outliers in terms of perceived threat. Further, we argue that this averaging, together with the randomized presentation of the stimuli, reduced possible effects of image features (e.g., eyes, color and spatial frequency) that differed between the two threatening conditions.

### Methodological Implications

As we have demonstrated in this study, the content of the pictures shown to the subjects has a pronounced effect on the neural response, even if the pictures are believed to be in the same emotion category (i.e., threatening pictures). As a consequence, we suggest that greater care should be taken when selecting stimuli for studies on emotion processing. In addition to the selection based on normative ratings, we recommend to characterize images also on qualitative dimensions, with the evolutionary-to-modern dimension being only one of several. For instance, Kensinger and Schacter (2006) had the subjects rate picture or word stimuli on the dimensions animacy (animate vs. inanimate) and commonality (common vs. uncommon). However, the authors report that the emotion processing did not differ depending on the task, whereupon the authors collapsed the data of both tasks. For a study containing a matching task and a labeling task, Hariri et al. (2003) used sets of threatening IAPS pictures which were virtually identical to our selection. Instead of evolutionary vs. modern, they denoted the stimuli being of natural vs. artificial origin. In the subsequent analysis, however, the authors collapse the fMRI data across these two different categories, since the focus of the study was the difference in task but not the differentiation of the two origins. In this case, pooling the data might cause undesired variance, assuming that the two categories engage the network of emotion processing differently.

From a more technical perspective, even measurable image parameters such as color, contrast, or spatial frequency do not directly account for the aspect of image content. Interestingly, also the RT did not differ between experimental conditions, giving no indication of a prioritized perception of evolutionary or modern threats (and thus, not reflecting our fMRI or behavioral results). Empirical evidence of this effect would suggest faster perception of threatening vs. neutral stimuli (Öhman et al., 2001) and no differences between evolutionary and modern threats (Fox et al., 2007; Pool et al., 2016).

In conclusion, exerting a more elaborate process of stimulus classification and selection will consequently lead to better experimental designs and thus more valid results. Effects that might have been confounded by the selection of overly heterogeneous stimuli could thus be revealed. We point out that researchers should be more aware of the possible effect of image content when selecting pictures as well as reporting results of studies using IAPS and comparable databases. We suggest that future studies utilizing affective picture stimuli should firstly replicate our findings of marked differences between evolutionary and modern stimuli, and secondly characterize the image content on more dimensions than only valence and arousal. The IAPS database was originally conceived on a theoretical foundation representing the basic emotion dimensions valence and arousal (Russell, 1980) and the dominance dimension. However, when applied in studies investigating neural processes, these dimensions might fall short of representing the complexity of the brain mechanisms adequately. Thus, adding further dimensions that are relevant and tailored to neurophysiological research might greatly improve the IAPS database and future studies. In addition, this study only investigated still pictures. However, real life visual perception is much more adapted to the perception of moving and animated scenes. Future studies might therefore also use short video clips to investigate effects of content on the processing of scenes.

### Limitations

The content selected for our experimental conditions could be criticized in different aspects. While both modern stimuli conditions show similar scenes from a wide-angle perspective, the modern-threatening condition also comprises close-up views of weapons. Also, the evolutionary-threatening pictures present menacing animals in their natural surroundings, whereas the evolutionary-neutral pictures do not contain any non-threatening animals. We are aware of this difference but argue that the inclusion of animals in both categories would have changed the focus to the comparison of animate vs. inanimate stimuli.

Similar to previous studies (Anders et al., 2008) and the original IAPS sample (Lang et al., 2008), we found that ratings varied significantly more on the arousal scale than on the valence scale. Anders et al. (2008) concluded that arousal ratings were thus less directly related to the actual stimulus than valence ratings. Moreover, the validity of the valence and arousal ratings should be cross-checked by other measures (e.g., verbal descriptions, thinking-aloud, etc.). The results are further to regard with the limitation that we did not match for physical properties of the pictures as luminance, color or complexity, as this would lead to very low samples of pictures with identical properties not suitable any more for statistically sufficient stimuli samples.

Furthermore, this study relies on subjective ratings of arousal combined with fMRI measures of brain activity. Psychophysiological measures such as heart rate, heart rate variability and skin conductance might increase the specificity of the findings.

Finally, we acknowledge that our findings and implications are not readily transferable to other basic emotions. While the effect of the evolutionary origin might be valid for threatening stimuli evoking fear or anxiety, it might not hold true for emotions such as happiness, disgust, sadness, or surprise.

## Conclusion

We provide evidence that neural activity in the fear module is not only driven by arousal or valence, but presumably also by the evolutionary content of the stimulus. Methodologically, we thus suggest that a more elaborate classification of stimulus content will improve the validity of experimental designs.

## Author Contributions

UH, ABB, MS, MD: substantial contributions to the conception or design of the work. UH, ABB, MS, SO, MD: acquisition, analysis, or interpretation of data for the work; final approval of the version to be published and agreement to be accountable for all aspects of the work in ensuring that questions related to the accuracy or integrity of any part of the work are appropriately investigated and resolved. UH, ABB, MS, SO: drafting the work (MD, UH) or revising it critically for important intellectual content. MD: prepared an earlier version of the manuscript, near to the current version with equivalent message and approved it. However, he then left the working group and did not contribute to the final revisions provided here. He was formally informed about the submission and did not contradict which is assumed as approval. Regarding his initial contribution, MD is still considered as first author.

## Conflict of Interest Statement

The authors declare that the research was conducted in the absence of any commercial or financial relationships that could be construed as a potential conflict of interest.

## Footnotes

^1^http://www.kek.zh.ch

^2^www.neurobs.com
