# Supplementary material for: Evolutionary and Modern Image Content Differentially Influence the Processing of Emotional Pictures
Source: Front Hum Neurosci. 2017 Aug 23;11:415. doi: 10.3389/fnhum.2017.00415 (PMC5572336; doi:10.3389/fnhum.2017.00415)
Supplement: Supplementary file 1 [file Table_1.docx]

Supplementary Material

**Evolutionary and modern image content differentially influence the processing of emotional pictures**

Matthias Dhum, Uwe Herwig, Sarah Opialla, Michael Siegrist, Annette B. Brühl

| 1026 |
| --- |
| 1030 |
| 1033 |
| 1040 |
| 1050 |
| 1051 |
| 1052 |
| 1070 |
| 5726 |
| 5750 |
| 5760 |
| 5780 |
| 5800 |
| 5811 |
| 5814 |
| 5825 |
| 6190 |
| 6230 |
| 6244 |
| 6300 |
| 7003 |
| 7017 |
| 7033 |
| 7036 |
| 7037 |
| 7039 |
| 7135 |
| 7140 |
| 9611 |
| 9901 |
| 9911 |
| 9920 |

Table S1 Number of IAPS pictures used for the experiment

|  | **MNI coordinates** | | |  |  |
| --- | --- | --- | --- | --- | --- |
| **Region (BA)** | ***x*** | ***y*** | ***z*** | **Cluster size (mm³)** | ***F_peak_*** |
| L middle frontal gyrus (9) | -42 | 16 | -1 | 197 | 14.31 |
| R inferior frontal gyrus (46) | 36 | 30 | 30 | 423 | 17.62 |
| R posterior cingulate gyrus (31) | 8 | -39 | 10 | 893 | 16.37 |
| R posterior cingulate gyrus (29) | 9 | -53 | 31 | 1384 | 42.74 |
| R cuneus (17) | 8 | -83 | 10 | 4742 | 54.31 |
| L occipital lobe, extending into the  inferior temporal lobe (18, 19, 37) | -43 | -82 | 5 | 54405 | 99.61 |
| R occipital lobe, extending into the  inferior temporal lobe (18, 19, 37) | 26 | -31 | -16 | 47724 | 75.84 |
| L amygdala | -26 | 4 | 3 | 1069 | 20.67 |
| R amygdala | 21 | 1 | -22 | 1145 | 21.37 |

Table S2 MNI coordinates of table 2; converted with: <http://sprout022.sprout.yale.edu/mni2tal/mni2tal.html>

|  | **MNI coordinates** | | |  |  |
| --- | --- | --- | --- | --- | --- |
| **Region (BA)** | ***x*** | ***y*** | ***z*** | **Cluster size (mm³)** | ***t*** |
| L inferior frontal gyrus (9) | -40 | 19 | -18 | 4022 | 5.67 |
| R middle frontal gyrus (9) | 37 | 11 | 25 | 623 | 5.21 |
| L fusiform gyrus (19) | -39 | -86 | 24 | 35262 | 13.36 |
| R fusiform gyrus (19) | 39 | -57 | -19 | 31408 | 12.46 |
| L superior parietal lobule (7) | -25 | -67 | -22 | 2195 | 5.16 |
| R superior parietal lobule (7) | 20 | -61 | 40 | 673 | 5.90 |
| R parietal lobe (sub-gyral) (7) | 23 | -57 | 76 | 400 | 4.93 |
| R precuneus (7) | 26 | -79 | 58 | 1827 | 5.51 |
| L amygdala | -30 | -6 | 46 | 1197 | 5.74 |
| R amygdala | 25 | 2 | -21 | 2815 | 6.42 |
| L thalamus | -22 | -27 | -18 | 434 | 6.19 |
| L posterior cingulate (30) | -22 | -56 | -4 | 2244 | -6.20 |
| R posterior cingulate (30) | 19 | -32 | 11 | 1641 | -7.15 |
| L parahippocampal gyrus (36) | -22 | -39 | -20 | 3359 | -8.79 |
| R parahippocampal gyrus (35) | 22 | -33 | -15 | 1775 | -8.04 |

Table S3 MNI coordinates of table 3; converted with: <http://sprout022.sprout.yale.edu/mni2tal/mni2tal.html>
